# WLTC and real-driving emissions for an autochthonous biofuel from wine-industry waste

**DOI:** 10.1038/s41598-021-87008-1

**Published:** 2021-04-06

**Authors:** Magín Lapuerta, José Rodríguez-Fernández, Ángel Ramos, David Donoso, Laureano Canoira

**Affiliations:** 1 Escuela Técnica Superior de Ingeniería Industrial, University of Castilla-La Mancha, Avda. Camilo José Cela, s/n, 13071 Ciudad Real, Spain; 2 Department of Energy & Fuels, ETS Ingenieros de Minas Y Energía, Universidad Politécnica de Madrid, Ríos Rosas 21, 28003 Madrid, Spain

**Keywords:** Environmental sciences, Energy science and technology, Engineering

## Abstract

Residues from the wine industry constitute an abundant feedstock for biodiesel production in wine-producing countries. The use of grapeseed oil, together with bioethanol obtained from distillation of wine surplus or grape skins and stalks and wine lees, as reagents in the transesterification reaction, results in a mixture of fatty acid ethyl esters (FAEE), which is a fully renewable, autochthonous, and waste-derived biofuel. In this work, a blend of FAEE produced from grape seed oil with diesel fuel was selected based on a study of fuel properties, and the optimal blend, with 30% v/v of FAEE, was tested in a Euro 6 engine following the Worldwide harmonized Light-duty Test Cycle (WLTC) and a Real Driving Emissions Cycle (RDE), as required in the new certification procedures. Engine performance and emissions from this blend and a commercial diesel fuel were compared. The FAEE blend showed a significant potential to reduce particle emissions, both in mass and number (from 23% in number to 46.5% in mass for WLTC, and from 56% in number to 61% in mass for RDE), and CO (25.5% for WLTC and 39% for RDE) but penalized NOx (32% higher in WLTC and 26.4% higher in RDE).

## Introduction

Recent studies have demonstrated that the use of organic waste as feedstock is the key point for sustainable, economic and environmentally friendly development of biofuels for transport^1^. In fact, Directive (EU) 2018/2001 displays a list of 17 feedstocks for the production of biogas and advanced liquid biofuels, which will be promoted towards a minimum share of 3.5% of energy consumption in the transport sector by 2030^2^. Among these 17 feedstocks, 14 of them are waste, either municipal, industrial, agricultural or livestock. Therefore, it is expected that waste-derived biofuels will play a preeminent role in the transport sector the next decade, together with renewable electricity and renewable liquid fuels of non-biological origin (known as power-to-liquid fuels or electrofuels)^3^.

Among all available sources for advanced biofuels, those produced abundantly in specific regions of the planet (autochthonous feedstock) are the most promising. Typically, autochthonous sources are cheaper and more sustainable than foreign alternatives, create job opportunities that contribute to local development and to stopping rural depopulation, diminish the global impact to biodiversity and strengthen energy security^4^. Nevertheless, the new pool of biofuels, regardless of their origin, will have to demonstrate the ability to reduce engine emissions and the compatibility with new aftertreatment systems when tested following the new certification procedures, which include the Worldwide harmonized Light-duty vehicle Test Cycle (WLTC) and the Real Driving Emissions (RDE) procedure.

Apart from grapeseed, produced in substantial amounts in regions such as Castilla-La Mancha in Spain^5^ or NAPA valley in California^6^, there are several others case studies of autochthonous sources for liquid biofuels. The use of fishery waste from New England was demonstrated as a cost-effective source for local biodiesel production^7^. Local varieties of castor plant have been grown in the northeast of Brazil for biodiesel production, using the residues for making biogas and biofertilizers^8^. Biodiesel from jatropha and karanja, non-food trees with low nutrient requirements and minimum care, represents an opportunity for rural and semi-arid regions in South Asia^9^, sub-Saharan Africa^10^ and India^11^. Local companies in the Mekong delta (Vietnam) plan to produce biofuel from catfish fat in large scale^12^. Also, waste oils from traditional sectors of the Turkish industry, such as leather manufacturing or fish processing, have been proposed^13^ and tested^14^ as biofuels for diesel engines. Local rice straw (Japan^15^), bagasse and other sugarcane waste (Iran^16^) or feedlot cattle manure in intensive livestock regions (Queensland and New South Wales, Australia^17^) are used and/or proposed as raw material in their respective bioethanol industry. Finally, studies about production and utilization of autochthonous biomass as solid fuel have been published^18,19^.

In the case of grapeseed, Spain has the largest vineyard area (1.0 Mha) in Europe and in the world, most of them located in the region of Castilla-La Mancha. In 2018, Spain was the second European grape producer (6.9 Mt) and the third wine producer (4440 ML)^20^. This substantial wine economy involves the generation of a large number of by-products, such as grape marc (15–20% respect grape weight) and wine lees (10–15% respect grape weight)^21^, which are included in the aforementioned directive^2^ as raw materials for advanced biofuels. Grapeseed oil is obtained from the seed, after separation from the grape marc^21^, whereas bioethanol can be not only renewable but waste-derived as far as it is produced by distillation of wine surplus^22^ and wine lees^23^, and also from stalks through more complex processes^24^. From the mentioned yearly production of grape and wine, the grapeseed oil production was 17.6 kt/year (4% of grape seeds with respect to grape weight and 6.4%w/w of oil content in grape seeds). The amount of FAEE produced could amount up to 15.9 kt/year, considering 90% yield.

There are previous experiences testing grapeseed-derived biofuels in diesel engines although the number is limited. Sreedhar et al.^25^ tested grapeseed biofuel blends on a single-cylinder DI diesel engine run under steady modes. They named this fuel as “biodiesel”, although it was not composed of alkyl esters but of smaller molecules resulting from the catalytic cracking of the oil. Lower NOx emissions were reported with the blends, but higher CO, THC and smoke, which indeed are results very different from the expectation for oxygenated biodiesel. However, results from Karthikeyan et al.^26^ and Sankar Ganesh et al.^27^ in non-modified single-cylinder engines confirm the NOx decrease and opacity increase with grapeseed oil methyl ester (GME) and its blends, with and without EGR. Later, the last authors^28^ optimized the bowl geometry (without modifying the compression ratio) of the original single-cylinder engine to improve the performance of diesel/GME blends. The combination of 25% GME blend and a deep bowl was found to increase the thermal efficiency by 5% compared to the standard piston and only-diesel operation, with a simultaneous reduction in smoke, CO and THC. Despite the low biodiesel content of this blend, NOx emissions increased up to 15% compared to diesel, which may be on account of the highly unsaturated nature of the biofuel^5,29^. Many works^30^ have demonstrated increasing NOx with the content of unsaturated esters, quantified through the iodine value. Moreover, NOx emission was particularly high when pure methyl and ethyl linoleate was tested pure^31^, being this compound the most abundant (around 70%) in grapeseed oil. Aware of this NOx increase, Vedagiri et al.^32^ performed tests analogous to those in^28^ with an emulsion of 5% of an aqueous solution of zinc oxide nano particles in grapeseed biodiesel. This emulsion decreased NOx up to 15% compared to pure grapeseed biodiesel, but the emissions were still a bit higher than that of pure diesel. To further reduce NOx emissions, the same authors^33^ tested grapeseed biodiesel with the above-referred emulsion in the same engine retrofitted with a SCR aftertreatment device, resulting in around 80% NOx reduction compared to diesel and no aftertreatment.

Grapeseed oil methyl ester is not a fully renewable fuel, because most of the methanol production is currently based on natural gas reforming^34^. This is not expected to change soon, since renewable alternatives for producing methanol^35^ will be counterbalanced by the increasing share of coal-derived methanol prompted from China^36^. In contrast, most of the worldwide ethanol industry is based on renewable and sustainable feedstock^37^, making grapeseed oil ethyl ester a fully renewable fuel. Despite the sustainable character of this biofuel, to the best of these authors’ knowledge no engine or vehicle tests have yet been published with grapeseed oil ethyl ester, pure or blended in diesel fuel. Furthermore, the reviewed tests with grapeseed oil-derived biofuels are limited to single-cylinder engines and steady conditions. In the present work, the tests are performed on an automotive diesel engine (equipped in current passenger cars) operated under the current certification transient cycle WLTC and with a real driving route compliant with the RDE procedure. This way, an autochthonous and fully renewable biofuel has been tested in an engine test bench simulating the driving and traffic conditions that are most typically encountered in the region where the feedstock is obtained and where the biofuel is produced. Finally, a life cycle assessment comparing the footprint of FAEE from grapeseed oil to biodiesel from palm oil (which has currently around 70% of the market share in Spain), has proved a significant reduction of CO_2_ emissions, resulting in 24.9 g CO_2_ equivalent per MJ of FAEE^38^. This significant reduction is mainly derived from 1) the use of an oil produced locally, avoiding long-distance transportation of the feedstock; 2) the different use of land, with grape production being destined to wine production while palm is grown as a biofuel crop; 3) the use of bioethanol from the wine production process, instead of methanol, avoiding the use of natural gas as input raw material.

## Experimental installation

**Figure 1:**
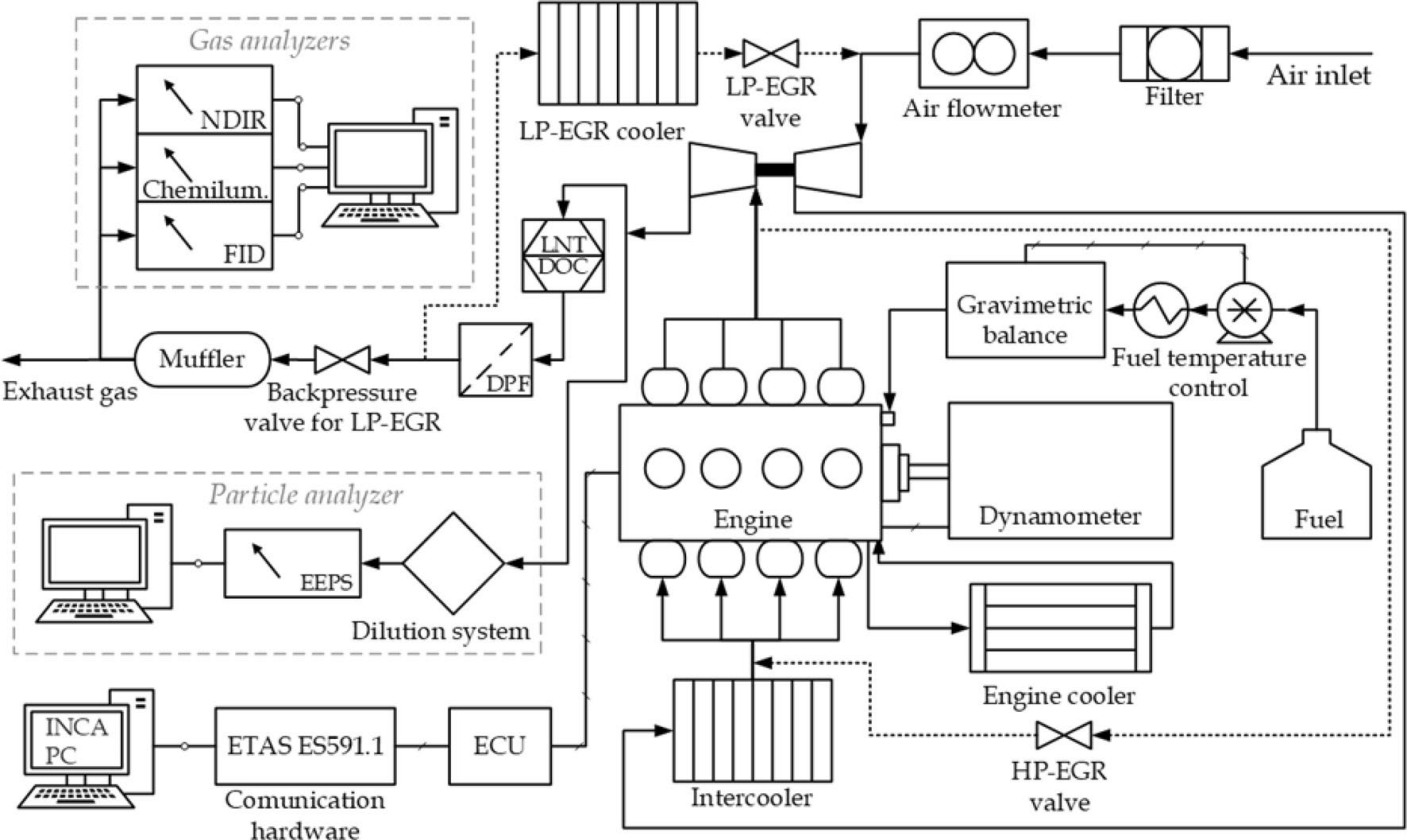
Experimental setup for engine tests.

The engine test bench used for the experiments is composed of an engine coupled to a dynamometer and exhaust gas and particle analyzers, as shown in Fig. 1. The engine is a common-rail direct injection diesel engine, with Euro 6 technology manufactured by Nissan, model K9K (1.5 dCi). The engine is equipped with double-loop exhaust gas recirculation system, low- and high-pressure (LP- and HP-EGR, respectively) which are managed according to the coolant and ambient temperature: LP-EGR for coolant temperature above 65 °C and ambient temperature above − 7 °C, and HP-EGR for coolant temperature below 65 °C regardless ambient temperature. The main specifications of the engine are summarized in Table 1.

**Table 1:** Engine specifications.

Cylinders	4 (in line)
Valves/Cylinder	2
Displacement (cm^3^)	1461
Stroke (mm)	80.5
Bore (mm)	76
Compression ratio	15.5:1
Injection	Common rail direct injection
Torque (max.)	260 Nm/1750–2500 rpm
Power (max.)	81 kW /4000 rpm
Aftertreatment system	DOC + LNT + DPF

The aftertreatment system is composed of a diesel oxidation catalyst (DOC), a diesel particle filter (DPF, wall-flow-type) and a lean NOx trap (LNT). Regardless the coolant and ambient temperatures, the HP-EGR system is also activated when the LNT purge is triggered.

To control the engine speed and torque, an asynchronous electric dynamometer (Schenk Dynas III LI 250) is coupled to the engine through the rotating shaft. A Road Load Simulation system (RLS, Horiba) was used to simulate transmission, tires, gearbox and other components of the dynamics of a Nissan Qashqai 1.5 dCi, which is one of the most popular sport utility vehicles (SUVs) in Europe. The main properties of the vehicle are shown in Table 2, including the resistance force ($F_{\left( V \right)}$, including rolling and aerodynamic resistances), as a function of the vehicle velocity ($V$), obtained from the coast-down procedure.

**Table 2:** Characteristics of the simulated vehicle.

Transmission	Manual, 6 gears
Differential ratio	4.13:1
1st:2nd:3rd:4th:5th:6th gear ratio	3.73:1; 1.95:1; 1.23:1; 0.84:1; 0.65:1; 0.56:1
Coast-down parameters*	$f_{0} = 89.6$; $f_{1} = 0.0659$;$f_{2} = 0.0391$
Vehicle test mass (kg)	1470

*$$F_{\left( V \right)} \left( {\text{N}} \right) = f_{0} + f_{1} V\left( {{\text{km}}/{\text{h}}} \right) + f_{2} V\left( {{\text{km}}/{\text{h}}} \right)^{2}.$$

The hardware ETAS ES 591.1 was used to communicate the INCA PC software and the electronic control unit (ECU), keeping the original settings of the vehicle mapping. Signals from raw sensors such as the air and fuel consumption (the latter previously calibrated with an AVL 733S fuel gravimetric system^39^) were registered with the INCA PC software.

Analysis of exhaust gases consists in measurements of total hydrocarbon (HC), carbon monoxide (CO), carbon dioxide (CO_2_) and nitrogen oxides (NOx = NO + NO_2_) emissions. CO and CO_2_ emissions were measured with a non-dispersive infrared technique (NDIR) in a MIR 2 M analyzer (with noise below 0.5%). HC emissions were measured with a flame ionization detector (FID) Graphite 52 M-D (with repeatability 1%), by pumping and filtering the sample at 190 °C. NOx emissions were measured using the chemiluminescence technique in a Topaze 3000 analyzer (also with repeatability 1%). These analyzers are integrated in a modular system from Envea SA. The modular system and the software allow performing calibrations and span tests. All gaseous emissions were measured downstream of the aftertreatment system.

The particle analyzer consists of a sampling system, which dilutes the gas sample from upstream of the DPF with a rotating disk diluter model MD19-2E (set at 150 °C to avoid hydrocarbon condensation) through a first dilution with a thermal conditioner model ASET15-4 (set at 300 °C), a second dilution system (a blending chamber which cools down the sample) and an Engine Exhaust Particle Sizer (EEPS) spectrometer model 3090 from TSI, which is able to measure the number and size of particles. The dilution factors for the rotating disk and thermal conditioner were 114.79:1 and 6.18:1 (values provided by the instrument manufacturer), respectively, leading a total dilution factor of 709.4:1. The dilution factor deserves special attention. For the RDE cycle, particle emissions are very high during some transient phases (high load sequences) compared to the rest of the cycle. This makes challenging to stablish an adequate dilution ratio to avoid over-ranged particle concentrations during these phases and to keep a good accuracy during the rest of the cycle. The particle mass was determined from the mobility diameter and particle number measured with EEPS, using to the density correlation proposed in^40^. Since the efficiency of particle filters is very high, all particle emission measurements were done upstream of the particle filter. At this sampling point, particle concentrations were very high, which explains such a high dilution factor.

This experimental installation has been described in^41^.

## Fuel selection

The fuels tested in this study were a first-fill commercial diesel fuel and a blend denoted hereinafter as GEE-30. This blend was composed of grapeseed oil-derived FAEE (30% v/v) and first-fill diesel fuel (70% v/v). The original grapeseed oil was donated by Movialsa (Spain) and grapeseed FAEE was produced as described in^5^. This FAEE content (30% v/v) was selected after measuring the main properties of blends with different contents. It was determined as the highest content that could guarantee not to experience filter clogging problems under cold ambient conditions at the engine start.

The first-fill diesel fuel was donated by Repsol. This type of diesel fuel has no biofuel content and has been used instead of conventional diesel fuel due to its low density, that allows GEE-30 to fulfill standard EN 590, despite the high density of the grapeseed-oil FAEE. Before blending, the grapeseed-oil FAEE was additivated with 1000 ppm of butylated hydroxytoluene (BHT) to improve the oxidation stability.

The main properties of the fuels tested (diesel and GEE-30) and the biofuel used in the blend tested (grapeseed oil FAEE) are shown in Table 3.

**Table 3:** Main properties of fuels tested or composing the blend tested.

Properties	Method	Diesel	Grapeseed-oil FAEE	GEE-30
Density at 15 °C (kg/m^3^)	EN ISO 3675	829.5	879.5	845.0
Kinematic viscosity at 40 °C (cSt)	EN ISO 3104	2.71	4.74	3.10
Lower heating value (MJ/kg)	UNE 51123	42.96	37.49	41.32
C (% w/w)	–	86.23	77.55	83.52
H (% w/w)	–	13.77	12.02	13.22
O (% w/w)	–	0	10.43	3.25
Fuel/air stoichiometric ratio	–	1/14.56	1/12.52	1/13.92
CFPP (°C)	EN 116	− 23.0	− 7.9	− 19.0
Cloud point (°C)	EN 23015	− 22.9	− 4.9	− 15.3
Pour point (°C)	ASTM D97	− 23.0	− 7.0	− 19.0
Lubricity (WS) (µm)	EN ISO 12156–1	443.1	219.6	303.9
Derived cetane number	EN 16715	58.61	50.77	54.70
Smoke Point (mm)	ASTM D 1322–97	22.4	39.5	27.3
Iodine number (g I_2_/100 g)	EN 16300	–	113	–
Distillation: T10 (°C)	EN 3405	228.5	320.6	244.7
T50 (°C)		262.0	–	287.4
T90 (°C)		298.9	–	343.6
Mean molecular formula	–	${\text{C}}_{13.55} {\text{H}}_{25.79}$	${\text{C}}_{19.81} {\text{H}}_{36.57} {\text{O}}_{2.00}$	${\text{C}}_{14.92} {\text{H}}_{28.14} {\text{O}}_{0.44}$

## Test cycles

Current certification procedures^42^ require the use of the WLTC driving cycle, longer (23.25 km) and much more dynamic than its predecessor, the NEDC cycle (with only 11 km). Besides, from September 2017, with the adoption of Euro 6d Standard, the WLTC cycle must be complemented in some cases with emission measurements in a RDE test, a trip carried out on public roads, open to normal traffic, trying to reduce the gap between the certified emissions and those measured under real driving conditions. RDE will apply to all new cars by 2021. A valid RDE trip must include urban, rural and motorway driving phases, defined exclusively on velocity intervals, and each of these phases must fulfill the specifications listed in Table 4. As shown in this table, the dynamic characteristics of the trip are controlled based on two parameters, the relative positive acceleration, *RPA* (i.e. the integral of the velocity multiplied by the time interval and the acceleration, if the last is positive, divided by the distance), for which a minimum is required to impede too soft trips and excessive steady-velocity periods, and the 95th percentile of the velocity-acceleration product, *v·a* (95) (a maximum limit is set as a function of the average velocity along the cycle, $\overline{v}$, to avoid too aggressive driving).

**Table 4:** Required specifications for RDE cycle and description of current RDE cycle.

	Urban	Rural	Motorway
**Distance (km), % of total distance (min–max)**			
Requirement	> 16, 29–43%	> 16, 24–43%	> 16, 24–43%
Current RDE cycle	26.8, 36.0%	23.0, 31.0%	24.5, 33.0%
**Velocity (km/h) (min–max)**			
Requirement	0–60	60–90	90–145
Current RDE cycle	0–53.3	21.6^a^–87.0	33.2^a^–122.2
**Average velocity (km/h)**			
Requirement	15–40	–	–
Current RDE cycle	24.3	74.5	105.5
**Stops (% of urban time)**			
Requirement	6–30%	–	–
Current RDE cycle	15.1%	0	0
**Time (s) at > 100 km/h**			
Requirement	–	–	> 300
Current RDE cycle	0	0	712
***RPA (m/s***^**2**^**)**			
Requirement	> − 0.0016 $\overline{v}$ + 0.1755, if $\overline{v}$ < 94 km/h > 0.025, if $\overline{v}$ > 94 km/h		
Current RDE cycle	0.2295	0.1162	0.0864
***v·a *(95) (m**^**2**^**/s**^**3**^**)**			
Requirement	< 0.136·$\overline{v}$ + 14.44, if $\overline{v}$ < 74.6 km/h < 0.0742·$\overline{v}$ + 18.966, if $\overline{v }$ > 74.6 km/h		
Current RDE cycle	10.74	14.58	16.75

^a^Values below the required limit in each phase correspond to a few instants for access to roads and roundabouts.

**Figure 2:**
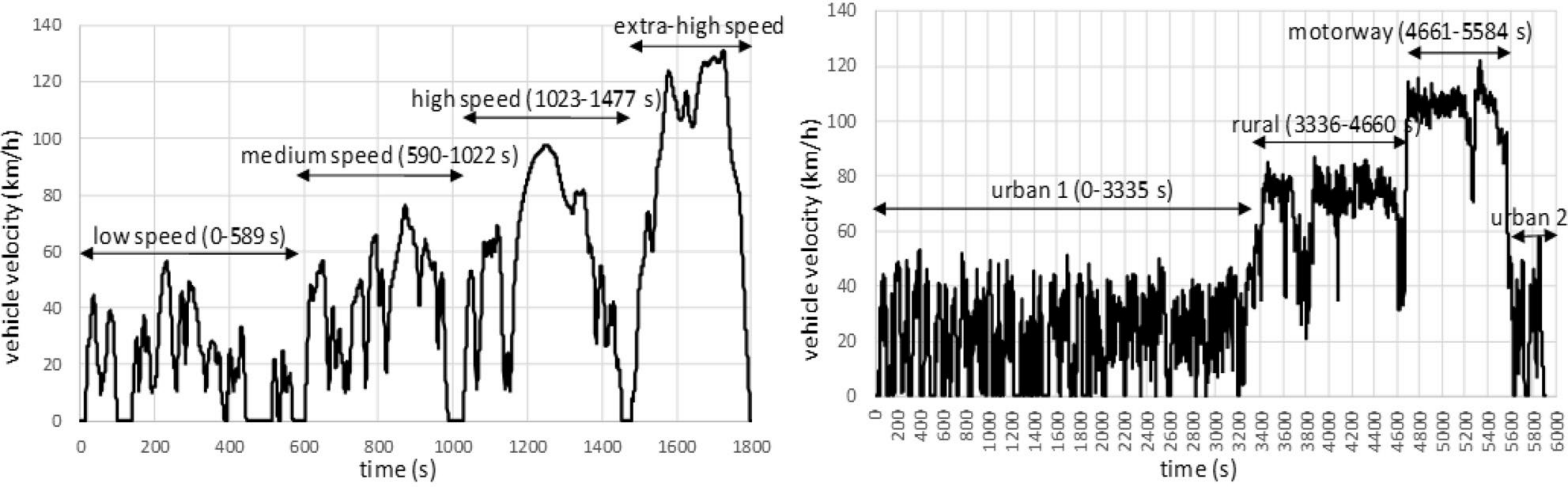
Driving cycles WLTC (left) and RDE (right).

The time-velocity-gear trace of the RDE simulated in the engine test bench with the Road Load Simulation system described in "Experimental installation" section was first acquired by driving the vehicle in Ciudad Real (Spain) and surroundings. The urban period was mostly covered in the city beltway and the university area, the rural period on the N-420 road from Ciudad Real to Daimiel (where velocities higher than 90 km/h are not allowed) and the motorway on the A-43 (from Daimiel back to Ciudad Real). The main metrics of the trip are shown in Table 4 to compare with the required specifications, and the velocity trace is shown in Fig. 2 along with the trace corresponding to WLTC cycle.

To analyze the results obtained in the RDE cycle, authors have divided the RDE cycle into 4 phases. The urban driving required in the specifications comprises the first and the last phases (named Urban 1, from the cold start to 3335 s, and Urban 2, from 5585 s to the end, i.e., 5915 s), while rural and motorway driving periods correspond to the second phase (Rural, from 3336 to 4660 s) and the third phase (Motorway, from 4661 to 5584 s). The last urban phase, although not required, was included to simulate a realistic go-and-return trip. The total distance amounted 74.3 km.

In all cases, ambient temperature remained at 23 °C ± 2 °C.

Prior to each test, a DPF regeneration and a LNT purge were triggered from the ECU software (to ensure the same aftertreatment conditions at the beginning of the tests), followed by at least 8 h soaking period. All tests were repeated at least three times. Average values and 90% confidence intervals were obtained for all instantaneous results and the confidence intervals were shadowed around the average accumulated values in the figures presented below, to distinguish the significance of the differences between diesel and GEE-30 fuels. Also, when mean results along the different phases of the cycles are presented, 90% confidence intervals are presented in the figures.

## Results and discussion

Fuel consumption (both instantaneous and accumulated), equivalence ratio, exhaust gas recirculation (EGR), were firstly analyzed for both driving cycles (WLTC and RDE), and then emissions were compared when the vehicle was fueled with diesel fuel and with GEE-30. Some of these parameters, such as equivalence ratio and EGR, are previously discussed because of their strong effect on the engine emissions, which must be considered to explain the effect of the fuel used.

### Fuel consumption

**Figure 3:**
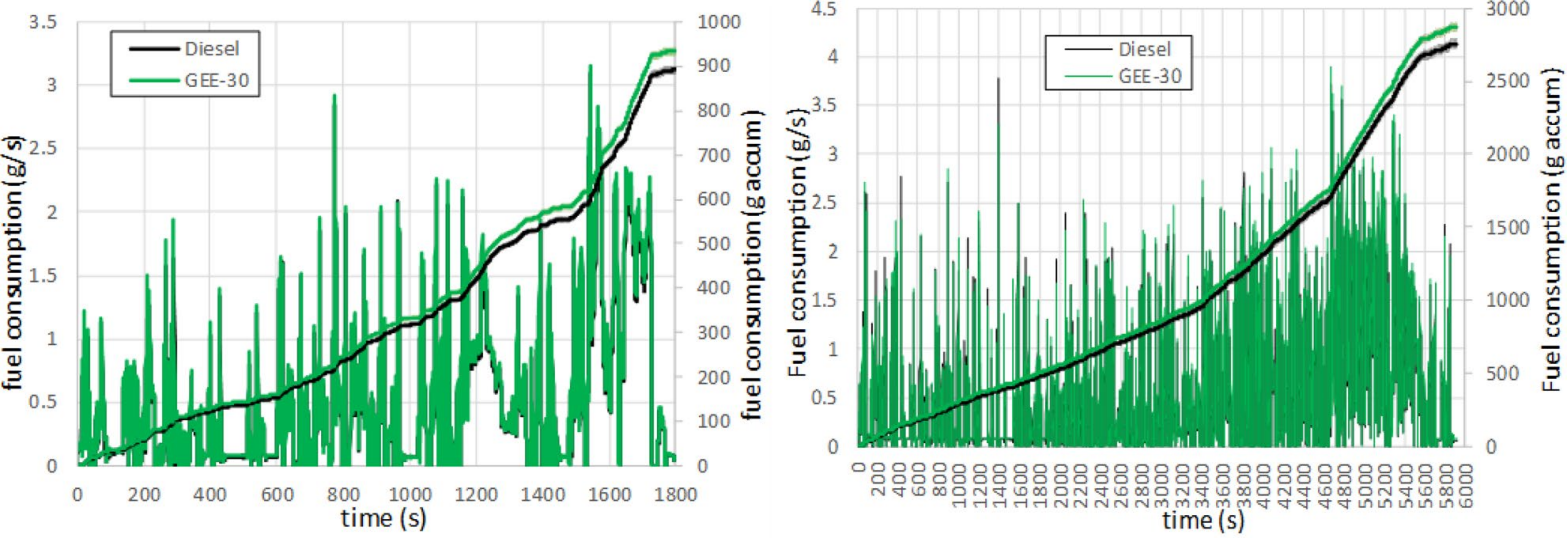
Instantaneous and accumulated fuel consumption along WLTC (left) and RDE (right).

The instantaneous and accumulated fuel consumption are shown in Fig. 3 for WLTC (left) and RDE (right) cycles, with very narrow confidence intervals. The peaks correspond to strong accelerations. Since GEE-30 has less heating value than diesel fuel, the amount of fuel injected is slightly higher, which leads to slightly higher fuel consumption at the end of the cycles. However, such increase is slightly lower in the RDE cycle (only 4.2%) than in the WLTC cycle (4.8%). Using the lower heating values (Table 3), the engine efficiency has been obtained for each phase and fuel. Results are shown in Figure S3 in the Supplementary Material. The mentioned differences in fuel consumption between fuels are compensated by the differences in lower heating value (3.8% lower for GEE-30 than for diesel fuel), leading to no significant differences in engine efficiencies, based on the confidence intervals.

**Figure 4:**
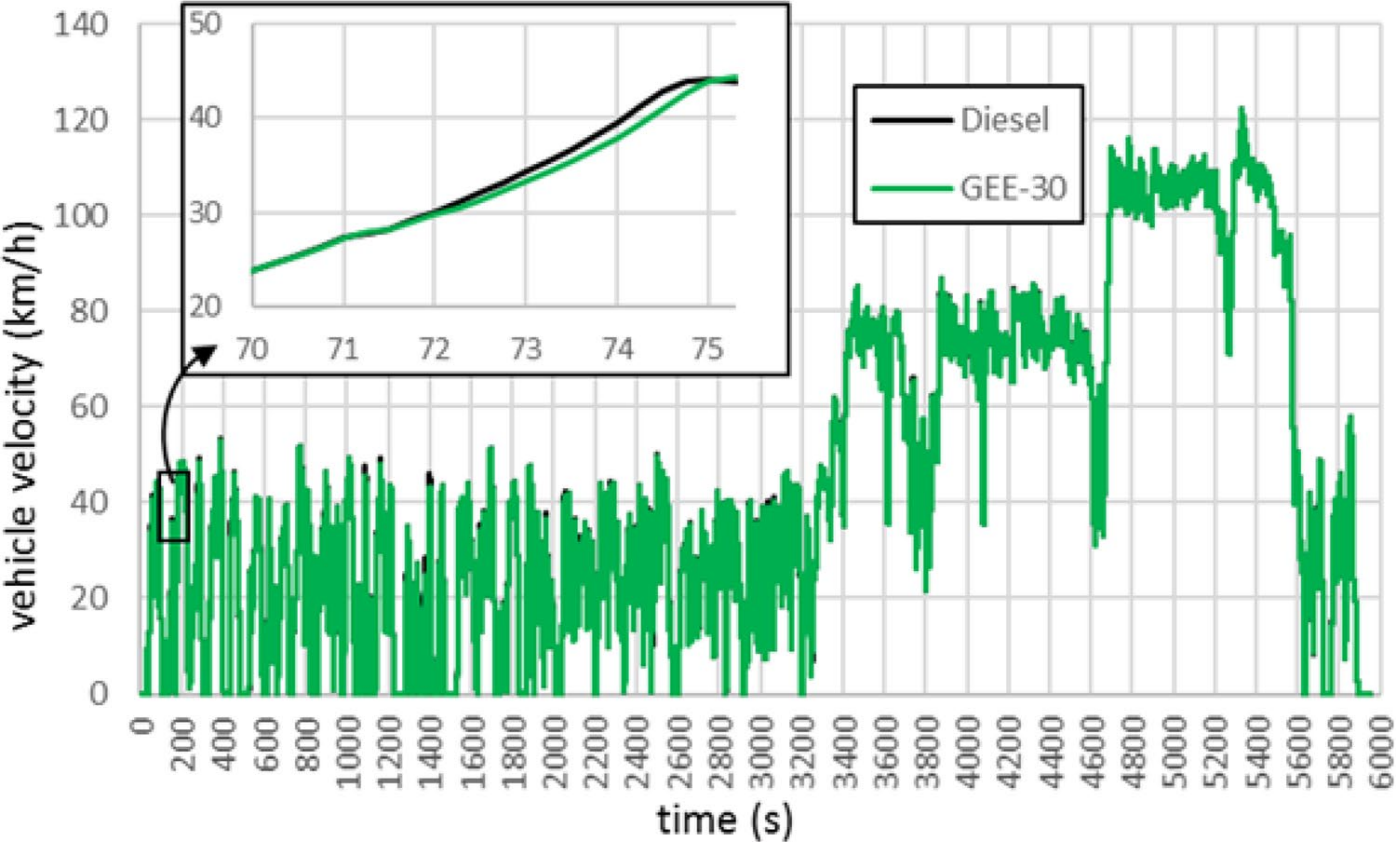
Detail on the vehicle velocity achieved during an acceleration in RDE.

Besides, although the velocity–time traces are defined and imposed equal for both fuels, slight differences are appreciable during full-load accelerations, which are more frequent in the RDE cycle (1.5% of the time in the WLTC and 3.7% in the RDE, these numbers have been obtained looking for accelerator positions higher than 90%). At full load, the injection system supplies the maximum fuel flowrate, which is the same (in volume units) for both fuels. This contributes to the observed lower difference in fuel consumption in the RDE cycle, at the expense of a certain power loss with the blend, and then, of a slower response in the velocity. An example of this is shown in Fig. 4 for an acceleration event in the urban phase of the RDE cycle.

### Equivalence ratio and exhaust gas recirculation

**Figure 5:**
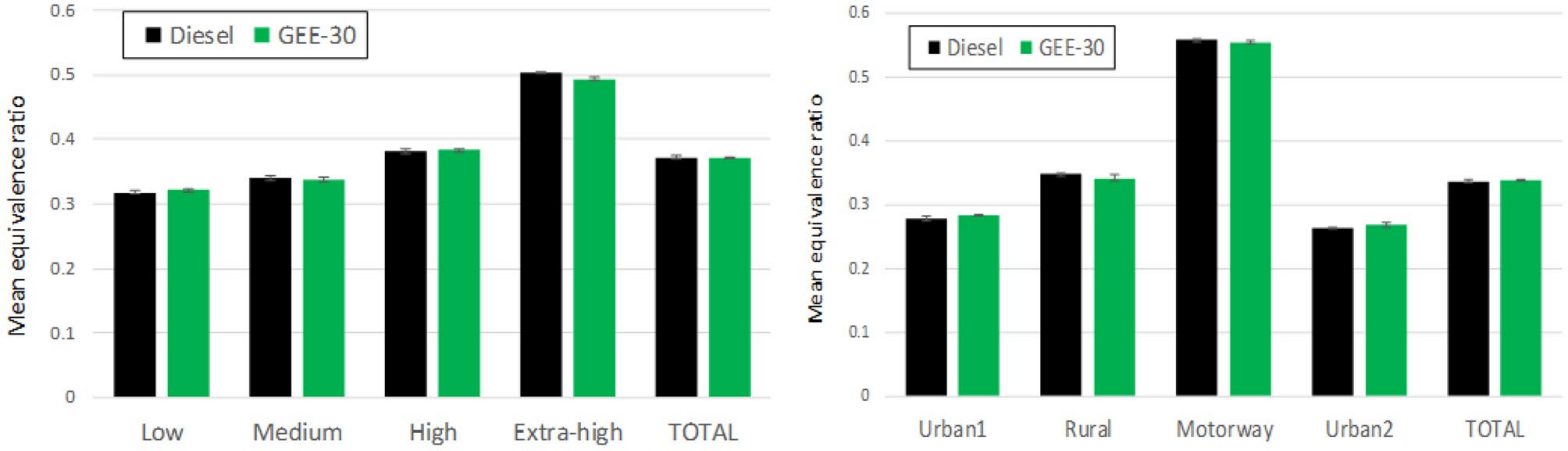
Mean equivalence ratio for the different phases of WLTC (left) and RDE (right).

The equivalence ratio is calculated dividing the instantaneous fuel/air mass ratio by the stoichiometric fuel/air ratio. The mean equivalence ratio is shown in Fig. 5 for the different phases composing each driving cycle. Each bar was calculated as the mean value of the average instantaneous values along each phase for each cycle. The average instantaneous values are shown in the Supplementary Material (Figure S1) for both diesel fuel and GEE-30 and for both driving cycles. As can be observed in Figure S1, the equivalence ratio reaches high instantaneous values both during accelerations and when de LNT regeneration becomes active (around 1215 s in WLTC and around 3425 s in RDE). The mean equivalence ratios shown in Fig. 5 are very repeatable from one test to another (very short confidence intervals). For both cycles, the type of fuel has no significant effect on the equivalence ratio because, although the instantaneous fuel/air mass ratio increases for GEE-30, the stoichiometric fuel/air ratio increases proportionally.

**Figure 6:**
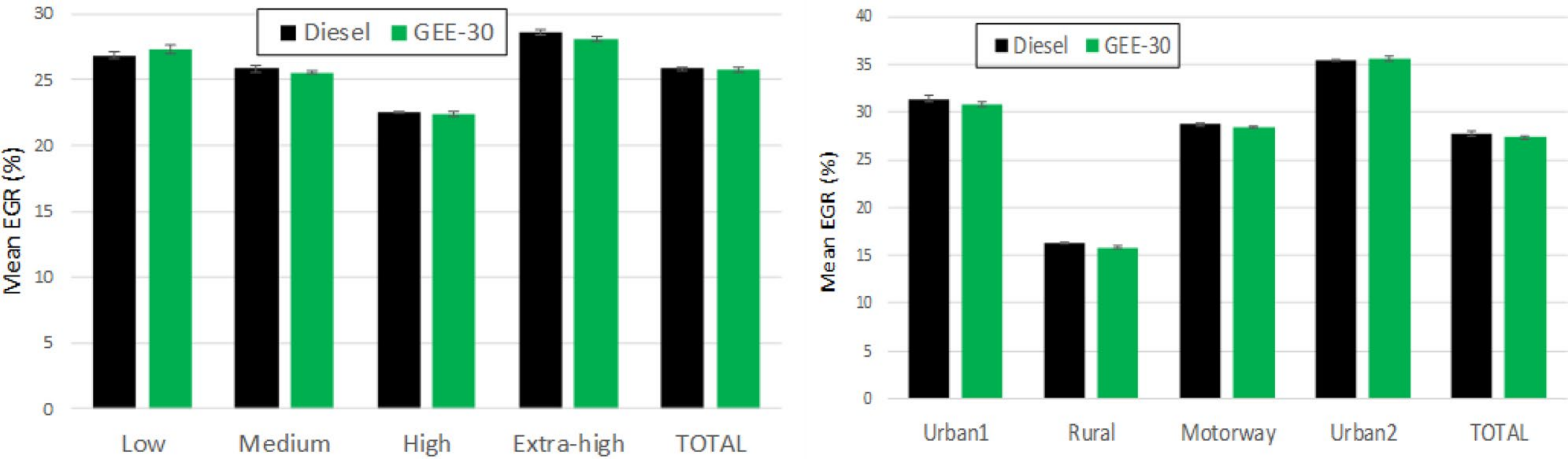
Mean EGR rate for the different phases of WLTC (left) and RDE (right).

The exhaust gas recirculation system is used to reduce NOx emissions. The EGR rate is one of the most sensitive parameters of the engine mapping. The high-pressure EGR (HP-EGR) or the low-pressure EGR (LP-EGR) are managed depending on some parameters such as the coolant temperature, the ambient temperature, the atmospheric pressure, the vehicle speed and the accelerator pedal position^43^. As observed in the mean values presented in Fig. 6, very small variations occurred among test repeats (short confidence intervals). This figure also shows (jointly with Figure S2 at the Supplementary Material for averaged instantaneous values) that the EGR rate remains basically unchanged for both fuels in both cycles.

From the comparison between equivalence ratio and EGR with both fuels (and in both driving cycles), it can be concluded that any difference observed in engine emissions should be attributed to the fuels, not to changes in equivalence ratio (which would affect CO, THC and particle emissions) or to changes in EGR (which would mainly affect NOx emission, but also to all other emissions).

### Gaseous emissions

As mentioned above, gaseous emissions were measured downstream of the after-treatment system and thus, the results depend on the efficiency of both the engine and the aftertreatment system.

**Figure 7:**
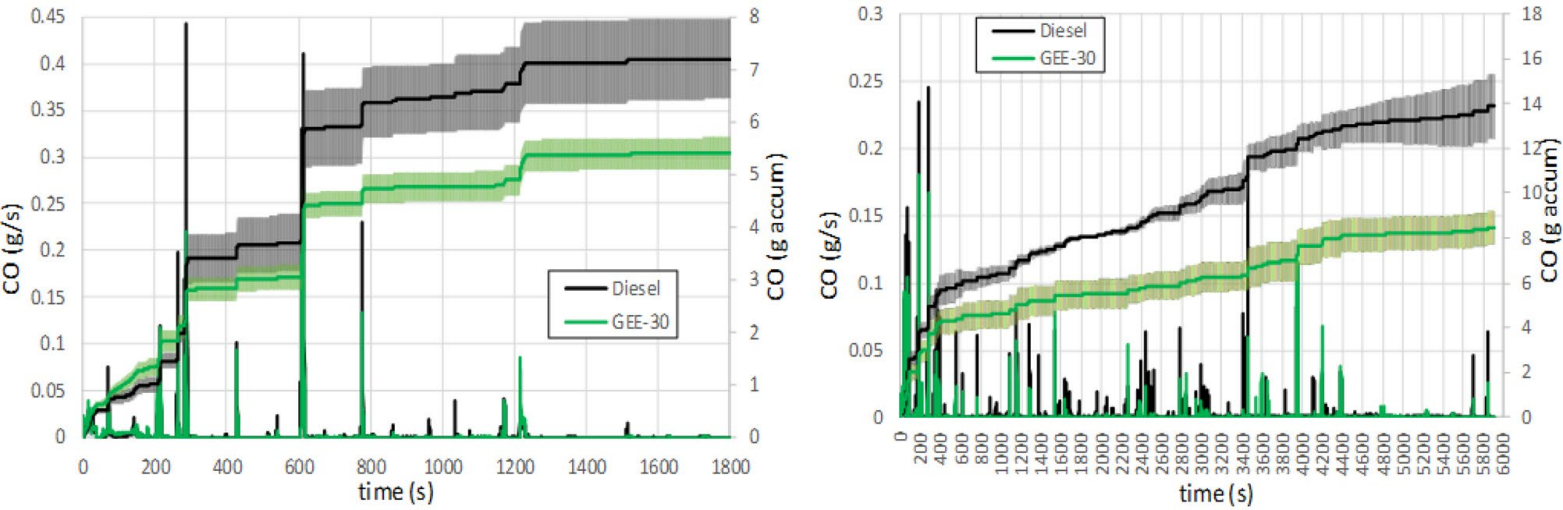
Instantaneous and accumulated CO emissions along WLTC (left) and RDE (right).

**Figure 8:**
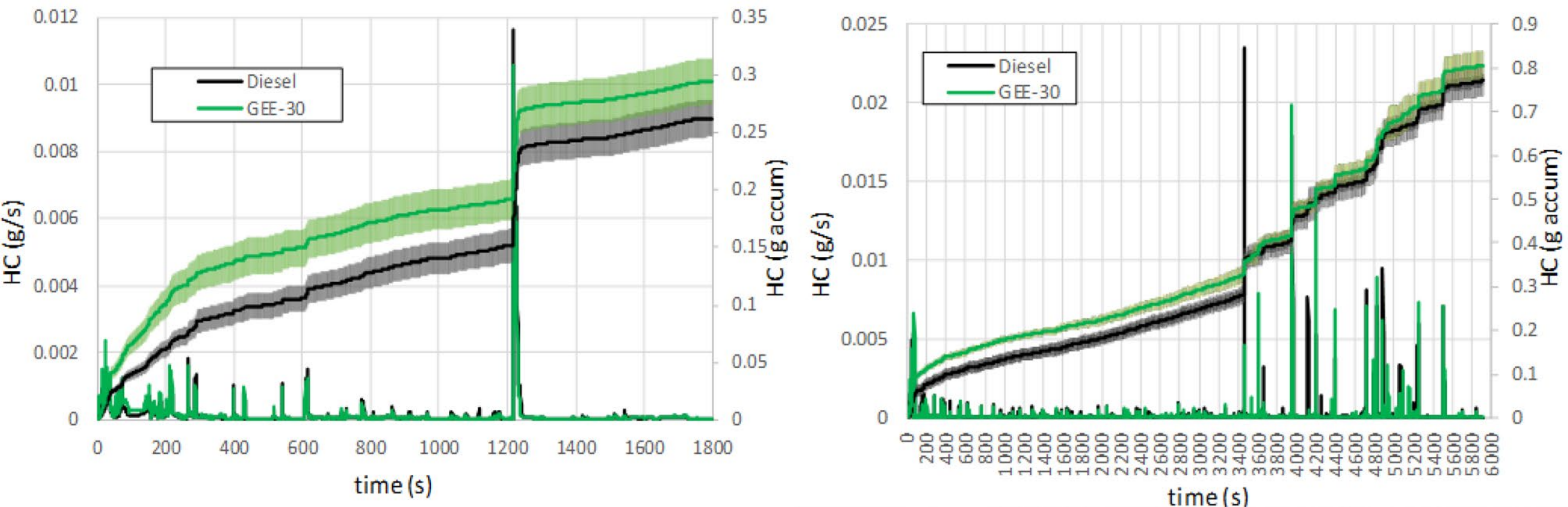
Instantaneous and accumulated THC emissions along WLTC (left) and RDE (right).

Figure 7 shows the mean instantaneous and accumulated CO along both driving cycles, WLTC and RDE. In both cases the peaks in CO emissions (and the corresponding increments in the accumulated curve) correspond to accelerations and LNT regeneration periods, in which the rapid increase in fuel injection suddenly increases the equivalence ratio. On the contrary, CO emissions remain very small during steady periods. They decrease as the exhaust temperature (see Figure S4 in the Supplementary Material) increase, as a consequence of the increase in the oxidation catalyst efficiency. The use of GEE-30 reduces CO emissions significantly (with no overlap between the confidence intervals at the end of the cycles) despite showing slightly higher emissions at the beginning of the cycles (under cold engine conditions). The final reduction in accumulated CO emissions is higher in the RDE cycle than in the WLTC one (39% in front of 25.5%) because the time with high load conditions is much larger in the RDE cycle. Hydrocarbon emissions (Fig. 8) have similar trends because they are also a consequence of incomplete combustion, and they are similarly affected by the low catalyst efficiency at the initial cold conditions. However, three main differences can be observed with respect to CO emissions: a) although the peaks observed in the figure also correspond to the accelerations, slight HC emissions remain even during non-acceleration periods; b) the peak corresponding to LNT regeneration is sharper with diesel fuel than with GEE-30 (a fuel post-injection producing an incomplete combustion leads to the necessary reducing atmosphere to get a good regeneration efficiency); c) the effect of cold engine operation at the beginning of the cycles is much more noticeable than in the case of CO emissions. The lower HC emissions observed with GEE-30 during the LNT regeneration tend to compensate the initial extra HC emissions. These lower HC emissions could be explained because of the higher HC consumption necessary for the reduction of higher amount of retained nitrogen dioxide in the Lean NOx trap during regeneration in case of using GEE-30 (consistently with the higher NOx emissions, as described below). At the end of both cycles, the confidence intervals overlap each other, especially in the case of RDE, but in general, the accumulated HC emissions are slightly higher in case of using GEE-30.

**Figure 9:**
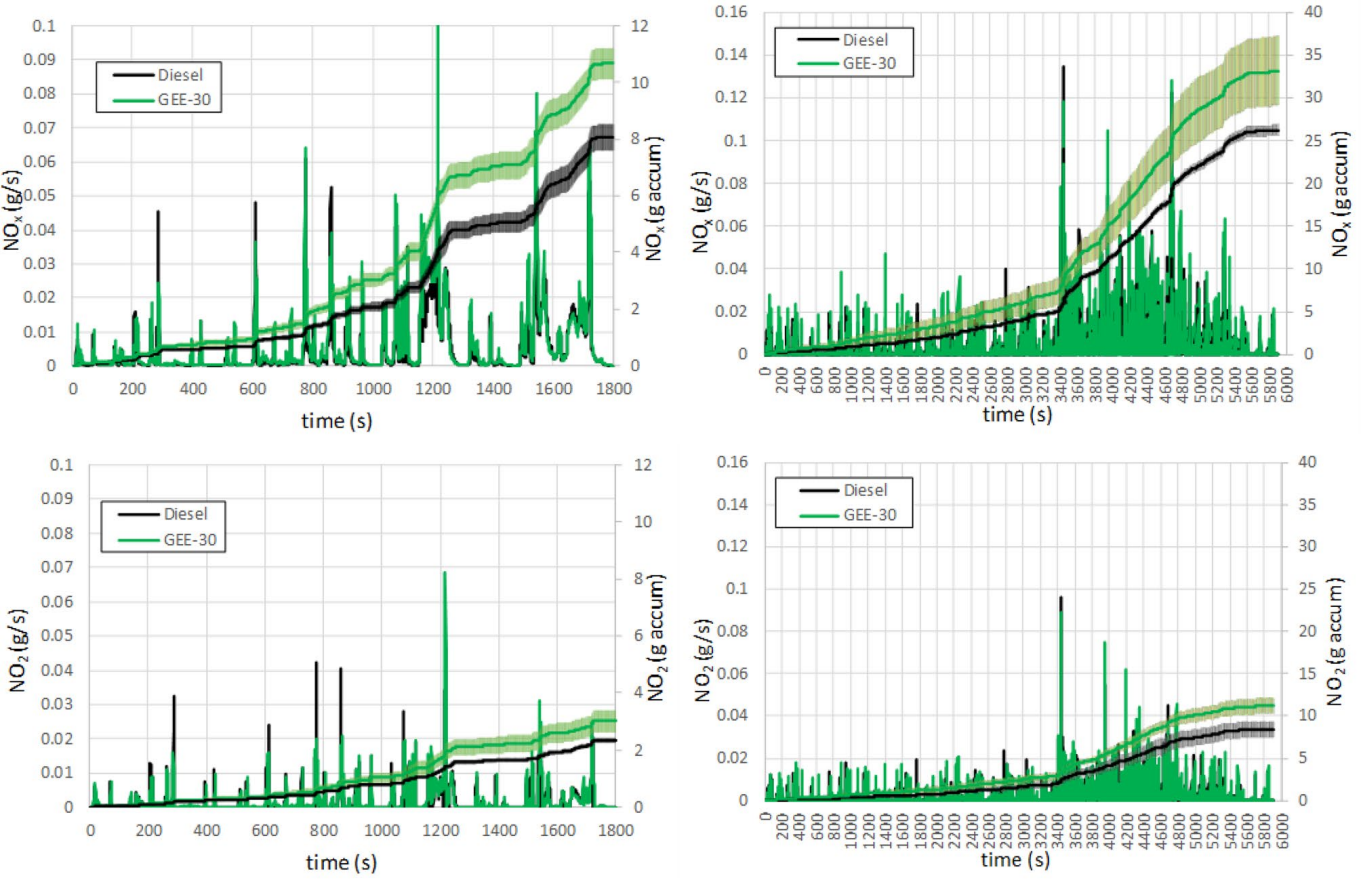
Instantaneous and accumulated NO_x_ (above) and NO_2_ (below) emissions along WLTC (left) and along RDE (right).

NO_x_ and NO_2_ (part of NOx) instantaneous and accumulated emissions are shown in Fig. 9 for both driving cycles. When the driving cycles operate under low load conditions more than half of the NOx emissions are NO_2_, but as the engine load increase, the fraction of nitrogen monoxide (NO) increase and the NO_2_ fraction decreases down to around one third for both fuels (29% in the case of WLTC and 33% in the case of RDE for both fuels). This demonstrates that at high cylinder temperature condition (see exhaust temperatures upstream of the turbine in Figure S4), the NO formation increases NOx emissions significantly. When fuels are compared, higher emissions are observed with GEE-30 (32% higher in WLTC and 26.4% higher in RDE). Such differences are small during the low speed phase, and start to be more significant (with higher rate of increase of NO_x_ emissions for GEE-30) in the medium and high speed phases. Similar trends can be observed for NO_2_ emissions. Among the reasons to explain these differences, the unsaturated nature of this ester (see iodine number in Table 3) could contribute to increase NO formation^44^ with respect to other biodiesel fuels.

### Particle emissions

**Figure 10:**
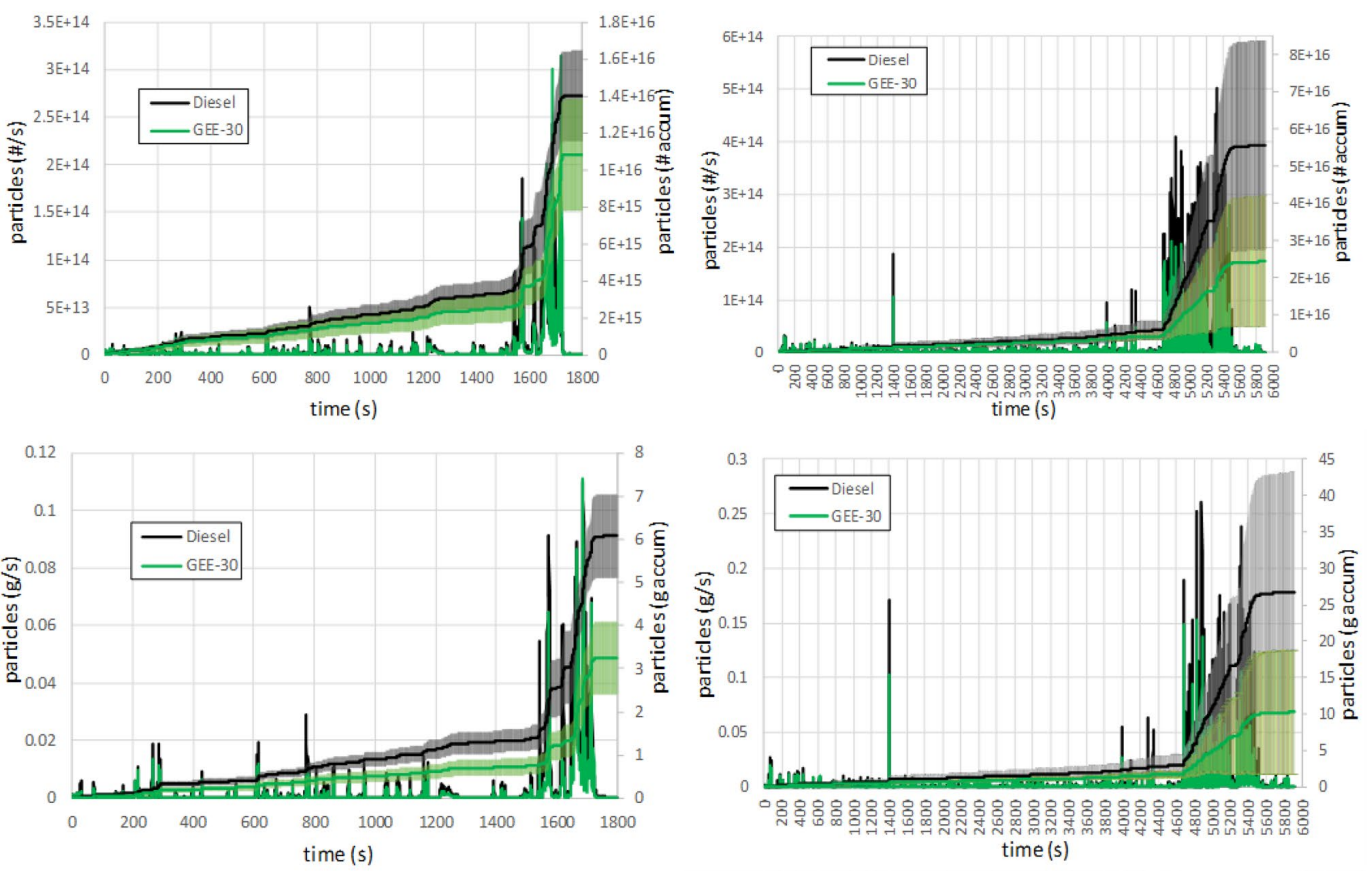
Instantaneous and accumulated particle number (above) and mass (below) emissions along WLTC (left) and along RDE (right).

Particle emissions were sampled upstream of the DPF in all tests. For a more complete information, although according to Regulation 2017/1151^42^ only particles larger than 23 nm should be counted, particles with diameters ranging from 5.6 to 560 nm were measured. The EEPS equipment provides the particulate number concentration as a function of diameter. The particle number emissions are shown in Fig. 10 (above panels) for both fuels and for both driving cycles. As can be observed, results were not very repeatable among different tests (large confidence intervals), as consequence of the high particle concentrations sampled upstream of the DPF. This effect was even more noticeable in the RDE cycle due to the high frequency of accelerations. Unfortunately, this effect reduces the significance of the results. The particle mass emissions are shown in Fig. 10 (below panels). These emissions were obtained by integrating the particulate size distribution and by using a size-density correlation^39^. Since they are derived from particle size distributions, wide confidence intervals (especially in the RDE cycle) remain here, although the overlap between intervals disappears in WLTC and is reduced in RDE.

Particle number and mass emissions depend on various factors, but the main one is the amount of oxygen in the combustion chamber. The oxygen available depends on the EGR rate, the equivalence ratio and the fuel composition. Since the two former factors did not show significant differences (see Figs. 5, 6), all differences should mainly be attributed to the characteristics of the fuels. In high load phases, and especially during accelerations, the engine operates under high equivalence ratios. At these conditions, the oxygen available in the combustion chamber is very limited and the oxidation process is hindered, increasing particle formation and emissions.

**Figure 11:**
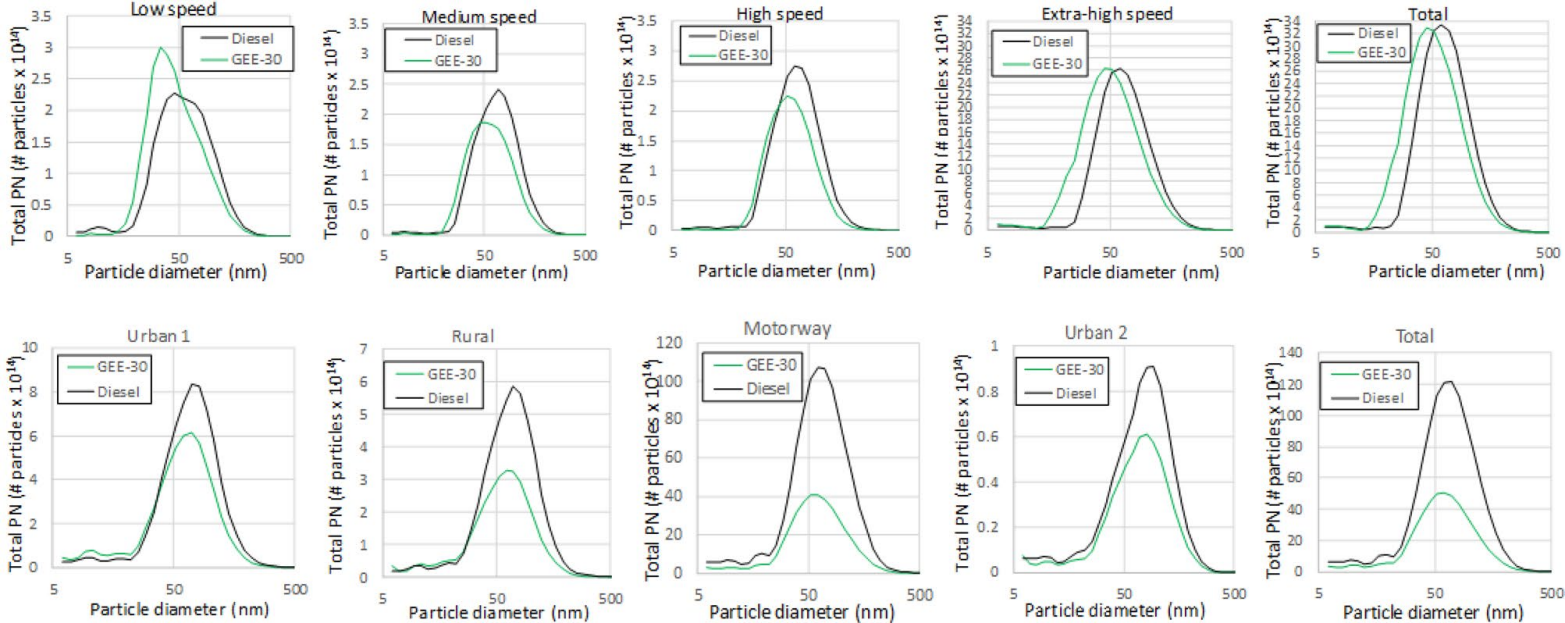
Average particle size distributions along WLTC (above) and along RDE (below).

**Figure 12:**
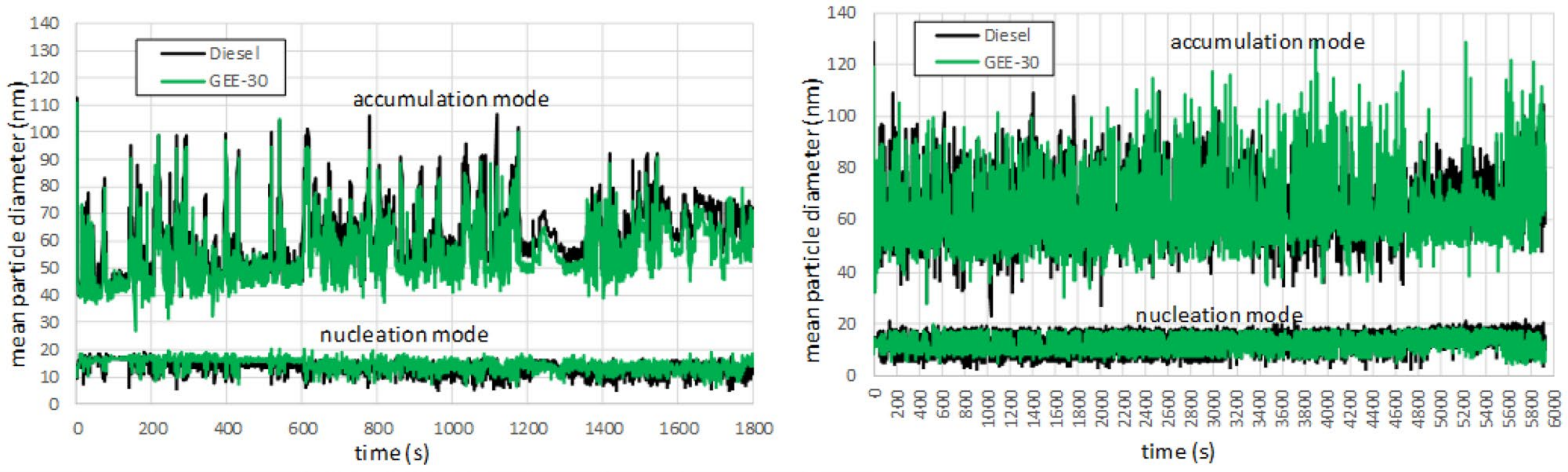
Mean particle diameters along WLTC (left) and along RDE (right).

Regarding the differences between fuels, GEE-30 shows a noticeable reduction in particle number emissions (although the confidence intervals slightly overlap each other as a consequence of the low repeatability of this measurement), and this reduction is even higher (and with no overlap between confidence intervals in the WLTC) in terms of particle mass (from 23% in number to 46.5% in mass for WLTC, and from 56% in number to 61% in mass for RDE). To explain this, average particle size distributions for the different phases of each driving cycle are shown in Fig. 11. As can be observed, particle size distributions are shifted towards smaller particles for GEE-30 at every phase in both cycles. This figure also shows that the number of particles is reduced with GEE-30 especially at high load, and this reduction is sharper in the RDE cycle, probably because of its higher transient character (much more frequent accelerations). The oxygenated composition of GEE-30 (3.25% as shown in Table 3) and its lower aromatic content contribute to the lower soot formation with respect to diesel fuel along the entire cycle, consistently with its higher smoke point (Table 3). The average diameters are shown in Fig. 12 for both nucleation mode (below 23 nm, and mainly composed of liquid hydrocarbon droplets) and accumulation mode (above 23 nm and mainly composed of soot agglomerates). As can be observed, average diameters in the accumulation mode from GEE-30 remain permanently below those from diesel fuel.

In summary, the use of GEE-30 instead of diesel fuel with the original engine calibration leads to benefits in PM emissions but slightly penalizes NOx emissions. Considering the well know trade-off between PM and NOx, it could be possible to slightly increase the EGR rate when the GEE-30 is used to reoptimize the engine calibration, avoiding any decrease in NOx emissions at the expense of reducing the benefits in particle emissions.

## Conclusions

A blend of grapeseed oil-derived FAEE (30% v/v) with first fill diesel (70% v/v), denoted as GEE-30, was tested in a Euro 6 engine test bench simulating the standard certification cycles, WLTC and RDE. The RDE cycle was defined by driving the vehicle in Ciudad Real (Spain) and surroundings. Grapeseed oil-derived FAEE is a fully renewable biofuel produced from waste of the wine industry and using bioethanol for the transesterification process.

Regarding the engine performance, an increase in fuel consumption can be observed with GEE-30 because of the reduced heating value. Although the velocity–time traces are imposed equal for both fuels, slight differences are appreciable during full-load accelerations, which are more frequent in the RDE cycle. This partially explains the slightly lower increase in fuel consumption observed with GEE-30 during the RDE cycle compared to the WLTC.

As expected, GEE-30 showed lower particle emissions (both number and mass) than diesel fuel. Since the equivalence ratio and the EGR rate did not show significant differences between fuels, the decrease in particle emissions with GEE-30 is mainly due to its chemical characteristics (higher oxygen and lower aromatic content). Such differences are more noticeable for particulate mass (rather than particle number) emissions because the particle size distributions are shifted towards smaller particles for GEE-30. Particle emission results in the RDE cycle show higher uncertainty, probably because particle formation is extremely high during the transient, high-load sequences included in the RDE. Particle formation under these conditions (close to stoichiometric ratio) is very sensitive to slight variations in the combustion chamber. Dealing with high particle concentrations and long tests is a current challenge for measuring techniques: high dilution ratios are the only solution to avoid saturation of the equipment at these conditions, but it compromises the accuracy of the measurement during the rest of the test.

High THC and CO emissions are mainly observed during the cold phase period before reaching the light-off temperature of the DOC. These emissions are manifested in the form of peaks during the rest of the cycle (attributed to accelerations and LNT regeneration process). The use of GEE-30 reduces CO emissions significantly despite showing slightly higher emissions at the beginning of the cycles during the cold phase. THC emissions shows similar trends, although the effect of the cold phase is much more noticeable than in the case of CO emissions.

GEE-30 shows higher NOx emissions but with significant influence of the phases. These differences are, in both cycles, small during the urban phases, and become more significant during the rest of the phases. Despite the NOx increased with GEE-30, the trade-off NOx-PM is still more beneficial for the blend. Therefore, there is margin in the engine calibration for increasing the EGR rate or modifying the injection parameters to decrease NOx emissions to the same level than those of diesel fuel while keeping some reduction in particulate matter emissions.

## Supplementary Information

Supplementary Information.
