# Supplementary material for: WLTC and real-driving emissions for an autochthonous biofuel from wine-industry waste
Source: Sci Rep. 2021 Apr 6;11:7528. doi: 10.1038/s41598-021-87008-1 (PMC8024388; doi:10.1038/s41598-021-87008-1)
Supplement: Supplementary file 1 — Supplementary Information. [file 41598_2021_87008_MOESM1_ESM.docx]

WLTC and real-driving emissions for an autochthonous biofuel from wine-industry waste

Magín Lapuerta^*1^, José Rodríguez-Fernández ^1^, Ángel Ramos ^1^, David Donoso ^1^, Laureano Canoira ^2^

1. Escuela Técnica Superior de Ingeniería Industrial, University of Castilla-La Mancha, Avda. Camilo José Cela, s/n, 13071 Ciudad Real, Spain
2. Department of Energy & Fuels, ETS Ingenieros de Minas y Energía, Universidad Politécnica de Madrid. Ríos Rosas 21, 28003 Madrid, Spain.

*e-mail (corresponding author): Magin.Lapuerta@uclm.es

**Supplementary Material**

*S.1. Instantaneous equivalence ratio and exhaust gas recirculation*


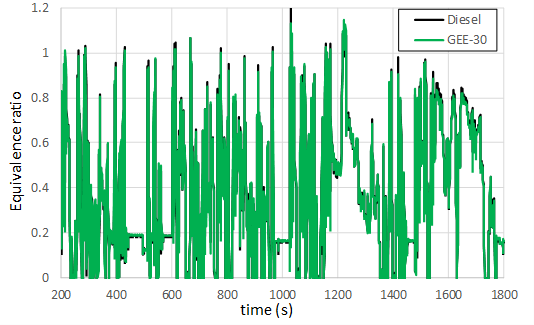

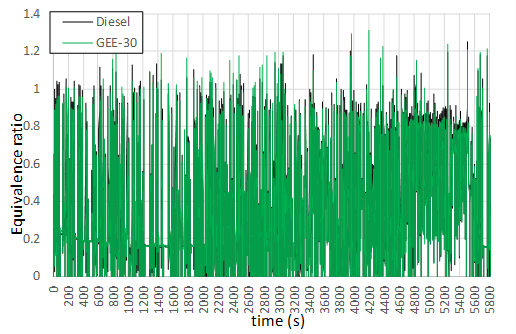


Figure S1. Instantaneous equivalence ratio for WLTC (left) and for RDE (right)


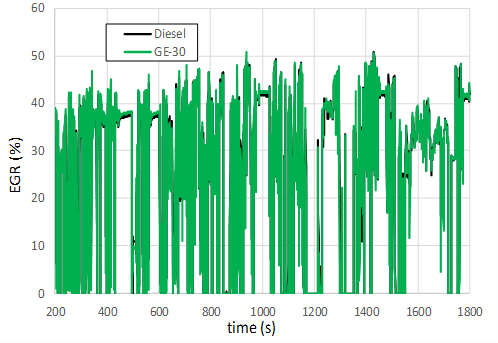

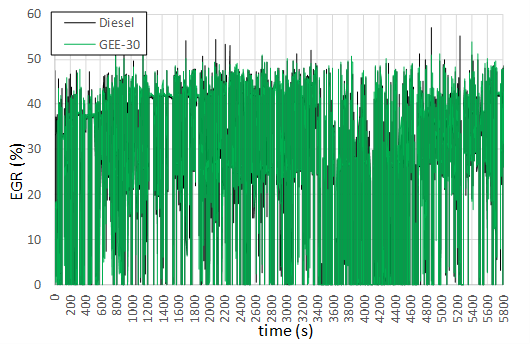


Figure S2. Instantaneous EGR rate for WLTC (left) and for RDE (right)

*S.1. Average engine efficiencies*

Figure S3. Average engine efficiency for WLTC (left) and for RDE (right)

*S.3. Instantaneous exhaust gas temperature upstream of the turbine*

Figure S4. Instantaneous exhaust temperature (upstream turbine) for WLTC (left) and for RDE (right)

Note: Contrary to all other results presented in this Supplementary Material, the exhaust temperature for cycle RDE for diesel fuel is plotted in front of that for GEE-30, to show that dispersion was lower for diesel fuel than for GEE-30 (Figure S3, right).
